# Watch, Imagine, Attempt: Motor Cortex Single-Unit Activity Reveals Context-Dependent Movement Encoding in Humans With Tetraplegia

**DOI:** 10.3389/fnhum.2018.00450

**Published:** 2018-11-15

**Authors:** Carlos E. Vargas-Irwin, Jessica M. Feldman, Brandon King, John D. Simeral, Brittany L. Sorice, Erin M. Oakley, Sydney S. Cash, Emad N. Eskandar, Gerhard M. Friehs, Leigh R. Hochberg, John P. Donoghue

**Affiliations:** ^1^Department of Neuroscience, Brown University, Providence, RI, United States; ^2^Robert J. and Nancy D. Carney Institute for Brain Science, Brown University, Providence, RI, United States; ^3^Center for Neurorestoration and Neurotechnology (CfNN), Rehabilitation R&D Service, Department of Veterans Affairs Medical Center, Providence, RI, United States; ^4^School of Engineering, Brown University, Providence, RI, United States; ^5^Center for Neurotechnology and Neurorecovery (CNTR), Department of Neurology, Massachusetts General Hospital, Boston, MA, United States; ^6^Department of Neurology, Harvard Medical School, Boston, MA, United States; ^7^Department of Neurosurgery, Harvard Medical School and Massachusetts General Hospital, Boston, MA, United States; ^8^Department of Neurosurgery, Rhode Island Hospital, Providence, RI, United States

**Keywords:** human, motor cortex, single unit, microelectrode array, tetraplegia

## Abstract

Planning and performing volitional movement engages widespread networks in the human brain, with motor cortex considered critical to the performance of skilled limb actions. Motor cortex is also engaged when actions are observed or imagined, but the manner in which ensembles of neurons represent these volitional states (VoSs) is unknown. Here we provide direct demonstration that observing, imagining or attempting action activates shared neural ensembles in human motor cortex. Two individuals with tetraplegia (due to brainstem stroke or amyotrophic lateral sclerosis, ALS) were verbally instructed to watch, imagine, or attempt reaching actions displayed on a computer screen. Neural activity in the precentral gyrus incorporated information about both cognitive state and movement kinematics; the three conditions presented overlapping but unique, statistically distinct activity patterns. These findings demonstrate that individual neurons in human motor cortex reflect information related to sensory inputs and VoS in addition to movement features, and are a key part of a broader network linking perception and cognition to action.

## Introduction

Beyond its central role in movement generation, primary motor cortex (MI) also appears to be engaged in cognitive and sensory processes in the absence of overt movement (Sanes and Donoghue, 2000; Hatsopoulos and Suminski, 2011). The earliest single neuron studies in non-human primates (NHPs) identified neurons in MI that increased their firing rates well before movement began, potentially reflecting memory or motor preparatory information (Tanji and Evarts, 1976; Georgopoulos et al., 1989; Riehle and Requin, 1989). Experiments in NHPs have also shown that observing an action can elicit MI activity both in local field potentials (Waldert et al., 2015) as well as single units (Wahnoun et al., 2006; Tkach et al., 2007; Dushanova and Donoghue, 2010; Vigneswaran et al., 2013). However, it is not possible to determine whether activity during action observation in NHPs reflects a passive perceptual process or a more cognitively driven “mental rehearsal.” Thus, these experiments do not resolve the issue whether motor cortex is representing action when there is no intention to act.

Perceptual and cognitive states are easier to influence in humans, since research participants can be specifically instructed to observe, imagine, or perform actions. Although fMRI studies have produced inconsistent results, there is increasing evidence for the activation of human MI across different perceptual or intentional states such as passive observation, imagined action, and movement performance (Gerardin et al., 2000; Dechent et al., 2004; Filimon et al., 2007, 2015; Sharma et al., 2008; Munzert et al., 2009; Macuga and Frey, 2012; Molenberghs et al., 2012). A majority of transcranial magnetic stimulation (TMS) studies also report that human motor cortex is more excitable during movement observation without any intention to act, suggesting that it is engaged during action perception and imagery (Fadiga et al., 1995; Aziz-Zadeh et al., 2002; Urgesi et al., 2006; Hétu et al., 2010; Naish et al., 2014). Additionally, Miller et al. (2010) used electrocorticography to demonstrate that the spatial distribution of high frequency field potentials (76–100 Hz) overlapped for motor imagery and execution in able-bodied human participants undergoing surgical treatment for medically refractory epilepsy. However, indirect methods cannot reliably distinguish whether these volitional state (VoS) effects reflect global excitability changes in motor cortex or the coordinated engagement of specific groups of neurons in MI (Dinstein et al., 2008). Addressing this question requires single neuron recording, which is rarely available for human motor cortex.

**Figure 1:**
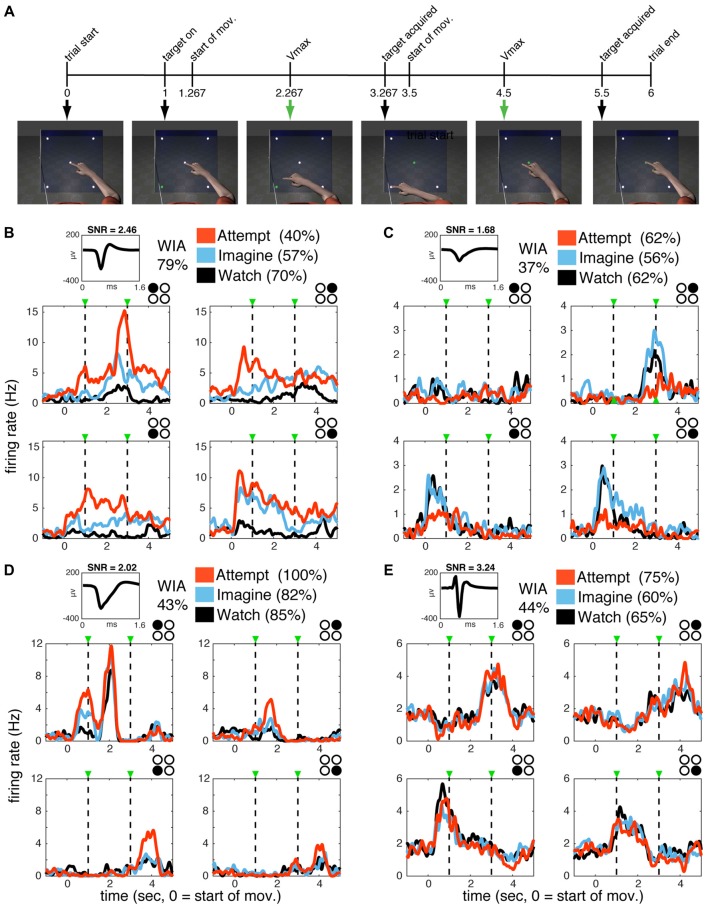
Single units in primary motor cortex (MI) are active during watch, imagine and attempt conditions. **(A)** WIA task sequence. The timeline of still frames indicates the time course of animated arm movements viewed by the participants, with 0 indicating movement onset. Event timing was identical for all movement directions. The same movies were utilized in different blocks paired with verbal instructions to watch, imagine, or attempt the displayed movements given before each block. **(B–E)** Single-unit activity for subject S3 **(B,C)** and T1 **(D,E)**. The mean waveform for each unit is shown on the top left of each panel. Classification accuracy for each unit (% of correctly classified trials) according to volitional state (VoS) condition or action (within each VoS condition) are listed on the top left (see “Materials and Methods” section for details). Mean firing rates across W, I and A conditions (averaged over ~10 trials for each target in each condition) are highlighted in black, blue and orange, respectively. Subplots are arranged spatially according to target position (indicated by filled black circle in top right corner). Plots are aligned to center-out movement onset (time zero). Dashed lines mark peak velocities for the center-out and return to center movements. Firing rates were calculated in 1 ms bins smoothed with a 250 ms Gaussian kernel for each neuron.

For the present study, the ongoing BrainGate2 clinical trial provided the ability to record ensembles of single neurons in the motor cortex of two humans during observed, imagined, and attempted reaching actions. Both participants, who had long-standing tetraplegia (due to brainstem stroke in participant S3 and amyotrophic lateral sclerosis (ALS) in participant T1), had a chronically implanted 96-channel microelectrode array (Cyberkinetics Neurotechnology Systems, Inc., and Blackrock Microsystems, Salt Lake City, UT, USA) in the hand/arm area of motor cortex (see “Materials and Methods” section for details). Participants were instructed to watch (W), imagine (I), or attempt (A) the actions displayed by an animated arm performing a four-target planar reaching task presented on a computer screen from a first-person perspective (“WIA task,” Figure 1A). Note that the same visual stimulus was provided across all conditions, so that the only difference was the participant’s degree of volitional intent (passive observation, mental rehearsal, or attempting to perform the action). Each trial of the animated movie sequence lasted 6 s and began with a 1 s baseline “stationary” epoch, during which the arm and all possible targets were visible and the index finger rested on the center target. One of the peripheral targets was illuminated at the end of the baseline period triggering the animated arm to begin a 2-s long reaching movement following a smooth, center-out trajectory toward the illuminated target. The index finger remained on the target for ~233 ms (7 frames at 30 frames/s), and then proceeded to return to the center target over the next 2 s. The arm remained in the final position for an additional 0.5 s after returning to the central target. The cycle was repeated with arm movements to randomly selected targets so that the upcoming action was unknown until the target light appeared.

## Results

### Volitional State and Action-Related Information in MI Neurons

We recorded three sessions with each of the two participants. Single units were identified using custom software (see “Materials and Methods” section for details). The total number of units identified across all sessions was 587 (115 in participant S3 and 472 in participant T1). The date (relative to array implantation surgery) and number of sorted units identified for each session are summarized in Table 1. Our experimental protocol included four different actions (videos of reaching movements aimed at four different targets) combined with three different instructions (W, I, A).

**Table 1:** Session information.

Participant	S3	S3	S3	T1	T1	T1
Post-implant day	1443	1452	1464	54	91	138
# of single units	28	37	50	137	159	176

For simplicity, we will refer to trials using the same video as having the same “action” (i.e., the full center center-out movement sequence, with distinct target goals and corresponding kinematics), and trials with the same instruction as having the same “VoS.” Note that the participants viewed the same set of four videos across all VoS conditions. The VoS condition varied across blocks for each session, while the movies (actions) were presented pseudo-randomly (see “Materials and Methods” section for details). Individual neurons displayed activity patterns that varied according to both VoS and action (Figures 1B–E). We used spike train similarity space analysis (SSIMS) in order to quantify the relationship between neural activity patterns observed across experimental conditions. The SSIMS algorithm is a relational encoding technique based on pair-wise similarity estimates between spike trains (taking into account the precise timing of each spike, in addition to the overall number of spikes recorded; Vargas-Irwin et al., 2015). This approach does not require an explicit model of the relationship between neural activity and external variables (e.g., a cosine tuning function for movement direction), and can detect a wider range of patterns than standard rate-based methods. Here, we use SSIMS to examine the relationship between different actions and VoS conditions, starting with representations for individual neurons, and then expanding to ensemble-wide activity patterns. We applied SSIMS analysis to single-unit spike trains spanning the center-out and return movements to the four different targets (corresponding to a 4.5 s window starting 200 ms after movement onset in the movie viewed by the participants). A neuron was considered to have significant action-related information if the distribution of SSIMS distances within a category had a significantly smaller median than those in different categories (Kruskal-Wallis *p* < 0.05). Stated another way, action-related information was detected if the neural data for trials with the same movie were more similar to each other than trials with different movies. The effects of VoS conditions were evaluated in a similar way.

**Figure 2:**
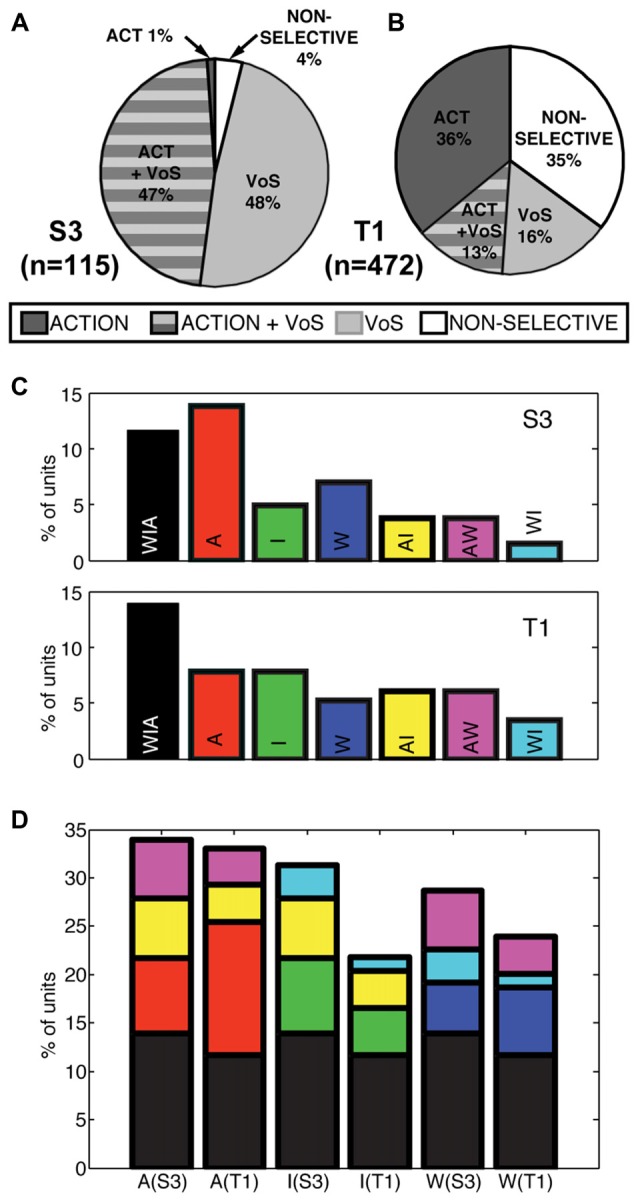
Actions and VoS are encoded by overlapping populations of single units.** (A,B)** Percentage of neurons displaying information related to VoS (watch, imagine, or attempt) or action (different movement) conditions in each participant (SSIMS analysis, see “Materials and Methods” section). The proportion of VoS related neurons is lower in T1, despite similar percentages of action related neurons. **(C)** Classes of action-related neurons, depending on engagement across W, I, A conditions (for example, yellow bars represent AI neurons, which displayed different activity patterns for movements aimed at different targets in both A and I). **(D)** Same bars and color scheme as in **(C)**, but with the bars stacked according to the types of neurons engaged in each VoS (separated by participant). The height of each bar represents the total percentage of action-related neurons recorded in each condition. Note that some of the stacked bars are shown more than once (for example, purple AW neurons are counted for both A and W conditions).

Single units displayed action, VoS and combined VoS+action information in both participants (Figures 2A,B). Overall, about one-half of the single units recorded displayed significant differences between movements to the four possible targets in at least one of the experimental conditions (S3 48%, T1 49%, Figures 2A,B). Neurons displaying VoS-related information, while readily evident in both participants, formed a substantially lower proportion of all recorded neurons in T1 compared to S3 (29%, vs. 96%), despite the fact that the total number of action neurons was nearly identical for both participants (Figures 2A,B). Nearly all movement-related neurons showed VoS effects in S3. By contrast, about one-third of movement cells showed VoS effects in T1. Units not selective for either direction or state were uncommon in S3 (4%) but accounted for more than one-third of neurons in T1.

### Interactions Between Action and Volitional State Information for Individual Neurons

We also evaluated whether action selectivity varied across VoS conditions. Overlapping sets of action-selective units were detected across W, I and A states (Figure 2C). Overall, approximately 25% of units displayed action-related information during W (S3 29%, T1 24%), ~24% during I (S3 31%, T1 22%), and ~33% during A (S3 34%, T1 33%). Overall, 26% of action-selective units were informative across all three VoS conditions (S3 28%, T1 25%) and ~22% were action selective across two VoS conditions (S3 31%, T1 20%). Accordingly, ~52% of action-selective units were specific to a single VoS condition (S3 41%, T1 55%).

**Figure 3:**
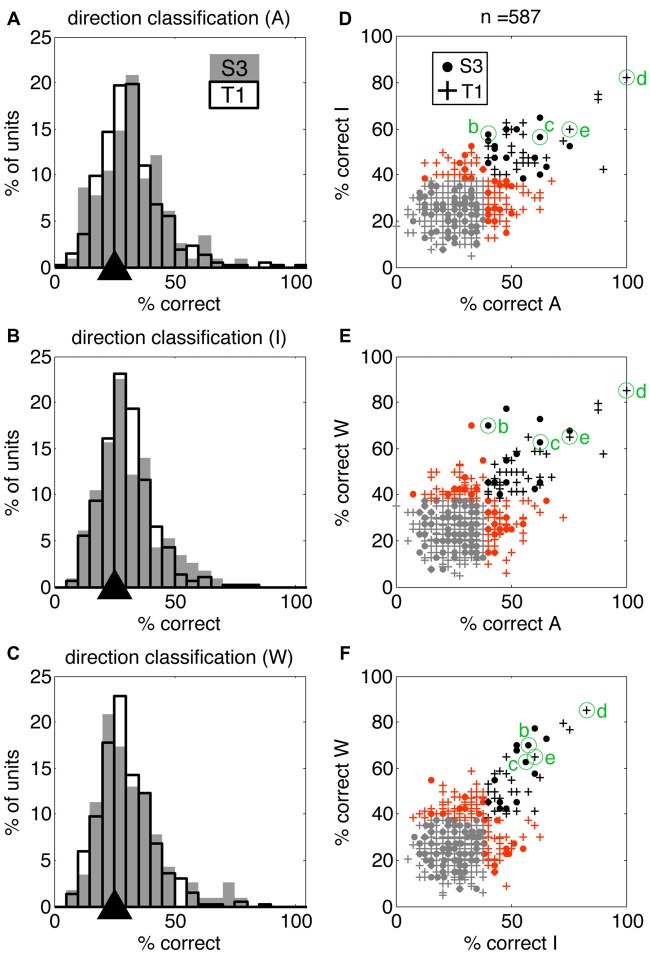
Single units display action-related information across volitional states. SSIMS NN classifiers were used to decode actions (reaching movements to four different targets) using single-unit data. **(A–C)** Distribution of single-unit classification results across neurons for each VoS, pooled across sessions for each participant. Gray bars correspond to participant S3, black outlines to participant T1. Black triangles on the *x*-axis mark the expected chance value. **(D–F)** Scatter plots comparing decoding performance across pairs of conditions. In each plot, units yielding classification below the 95% confidence limit of the chance distribution are shown in gray, units over the 95% confidence limit in only one category shown in orange, and units yielding significant decoding in two categories are shown in black. Filled circles correspond to units from participant S3, while + signs are used for units from participant T1. The units labeled **(B–E)** correspond to those shown in Figure 1.

We quantified information present in individual neurons using discrete nearest neighbor classifiers to decode the four reaching actions presented in the movies (using the same 4.5 s windows; see “Materials and Methods” section for details). This approach allowed us to test if individual neurons contributed to action encoding to a different extent across VoSs by comparing decoding accuracy in each VoS condition. It was possible to classify the four action conditions above chance levels for all three states in both participants using single-unit information (Figures 3A–C). Neurons providing highest decoding accuracy (>60%) tended to be informative across multiple conditions, as shown by clustering across the diagonals in Figures 3D–F.

### Ensemble Decoding

**Figure 4:**
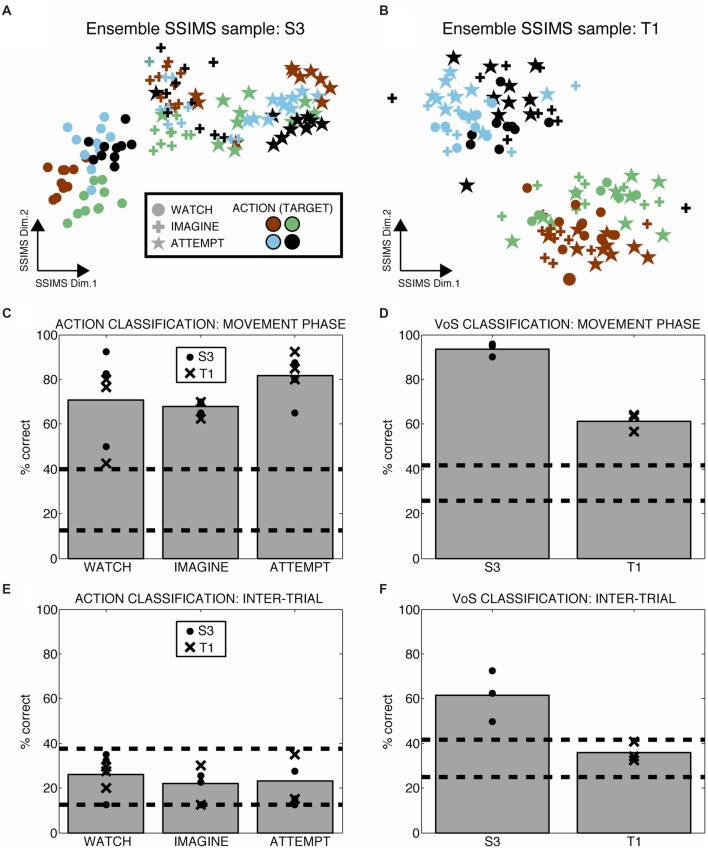
Ensemble firing patterns reflect action as well as volitional states. **(A,B)** 2D SSIMS plots for one sample session in each participant. Each point represents the activity of the entire simultaneously recorded ensemble during one trial. The distance between points represents the similarity between the ensemble-level spiking patterns observed. Symbols denote W, I, A condition and colors denote reach target position, as shown in the key. Clustering of similar symbols denotes similarity between trials in the same VoS condition, while clustering of similar colors denotes similarity between trials with the action (movie). **(C,D)** Action and VoS decoding results for each session. Dashed lines represent the 95% confidence interval of the chance distribution (obtained empirically from 10,000 random shuffles of trial labels). **(E,F)** Similar plots to **(C,D)**, except using 0.5 s of data recorded before the presentation of each movie (inter-trial interval, ITI). Only VoS decoding in participant S3 was above chance levels during the ITI.

Heterogeneous ensembles of the single units described so far were simultaneously engaged during the WIA task. We applied ensemble-level SSIMS analysis to evaluate the information emerging at the population level on a trial-by-trial basis (ensemble activity is represented by concatenating the similarity vectors for individual neurons, as described in Vargas-Irwin et al., 2015). This approach allows the firing pattern of the entire ensemble of simultaneously recorded neurons to be projected as a single point in the SSIMS representation. While the data exist in high dimensional space, it is possible to visualize the relationships between activity patterns using two-dimensional SSIMS plots (Figures 4A,B) The relative position of individual trials in the SSIMS projections is solely determined by the inherent similarity in the spiking activity across all neurons. In agreement with our single-unit results, clustering according to VoS was most evident in participant S3, while the action category dominated the relationships between neural activity patterns in participant T1. In order to quantify this pattern, we performed VoS and action classification using ensemble SSIMS projections. Classification was performed in a 15D SSIMS space in order to capture information not directly represented in the 2D plots used for visualization (increasing dimensionality further did not improve decoding accuracy). Ensemble activity patterns were sufficiently different to reliably classify both action and VoS above chance levels in every recorded session (Figures 4C,D). Although action decoding was similar between the two participants, VoS decoding accuracy was substantially better for participant S3 (61 vs. 94%).

We tested whether the different instructions affected neural activity outside of the periods where the movies were presented by evaluating ensemble decoding during the inter-trial interval (ITI, the time in between the presentation of individual movies). Action decoding in the ITI (before any direction information was available) was within the expected chance levels for both participants (Figure 4E). Although VoS decoding during the ITI was within the expected chance limits for participant T1, we observed significant decoding in participant S3 (Figure 4F). Given the block design used for the WIA task instructions, the participants had advanced information about the type of trial before action was displayed in the movie. Our results suggest that different baseline activity patterns reflecting VoS for participant S3 were evident even before the movies were presented.

### Neural Response Latency

**Figure 5:**
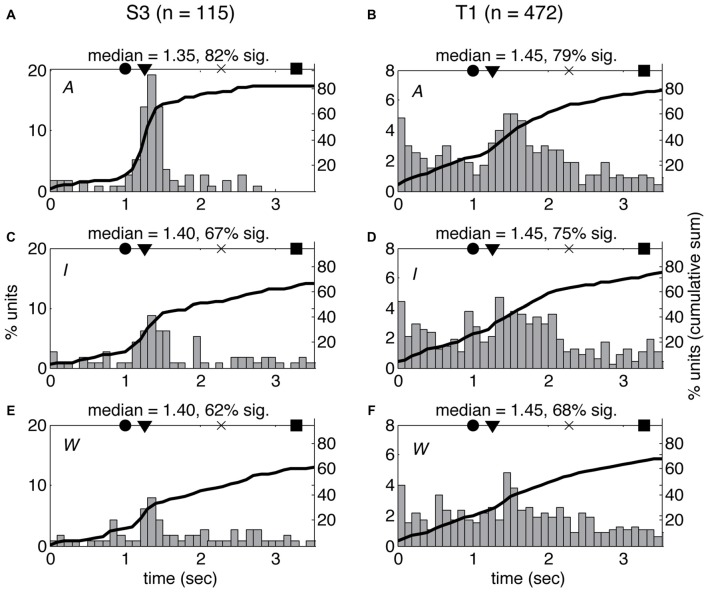
Latency of neural response does not vary across volitional states. Histogram of single-unit response latencies (occurrence of first significant change in firing rate, see “Materials and Methods” section) for both participants (S3: **A**,**C**,**E**; T1: **B**,**D**,**F**) across the three VoS conditions (attempt: **A**,**B**; imagine: **C**,**D**; watch: **E**,**F**). Time zero corresponds to the beginning of the movies. Circles denote the time of target illumination, triangles movement onset, × maximum velocity and squares target acquisition. Black line shows the cumulative sum of the percentage of neurons displaying significant changes in firing rate at each time point.

The differences in spiking patterns observed between the three VoSs could reflect variations in the overall timing of the neural response for observed, imagined and attempted movements. In order to test this possibility, we examined the latency of the first significant change in firing rate for each unit between the beginning of each movie and the end of the first movement using a SChI (similar to Rao and Donoghue, 2014, see “Materials and Methods” section). The estimated median latencies ranged between 1.35 s and 1.45 s from the first frame of the movie, which shows the arm at rest with the finger on the center target (corresponding to 83–183 ms after the first frame showing movement). There was no significant difference in the median latencies observed for W, I, and A conditions in either of the participants (KS *p* > 0.05). However, more neurons showed significant firing rate changes for the time period examined in A compared to I and W (Figure 5). There was no significant difference between the median latencies observed across participants (KS *p* > 0.05).

### Overall Changes in Cortical Excitability

**Figure 6:**
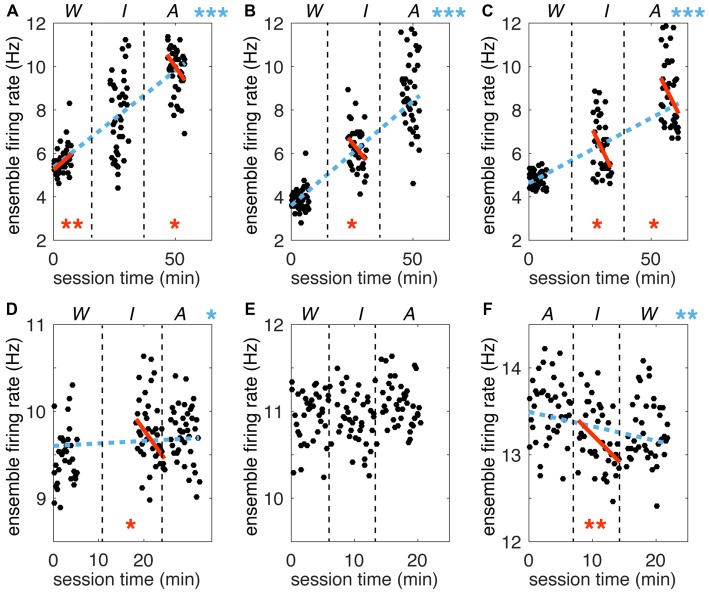
Trends in ensemble firing rate across time. Ensemble firing rates (EFRs) for each trial (mean across all simultaneously recorded neurons) are plotted against total session time. **(A–C)** Sessions for participant S3. **(D–F)** Sessions for participant T1. Vertical dashed black lines separate VoS condition blocks, which are labeled above each plot (note reversed order of sessions in **F**). Dashed blue lines represent the Theil-Sen slope estimate for the full session. Solid orange lines represent the slope calculated for each W, I and A block separately. Only slopes significantly different form zero are shown (Kendall’s Tau). Asterisks are used to indicate *p* values (*p < 0.05; **p < 0.01; ***p < 0.0001).

A gradual increase in cortical excitability through time could result in different activity patterns across blocks. This effect could potentially account for apparent changes linked to VoS. The fact that simultaneously recorded neurons individually could show opposite trends or no change across blocks diminishes the likelihood of this explanation. In order to evaluate overall changes in excitability, we tracked the total number of spikes detected during each trial for each of the six recording sessions. We calculated ensemble firing rates (EFRs) by taking the average firing rate across all recorded neurons for each trial over the 4.5 s analysis window encompassing center-out and return movements. Although baseline EFR varied from day to day, values were consistently higher in the A condition, followed by I, and lastly W. We used a Theil-Sen estimator (Wilcox, 2010) to quantify changes in EFR over time (Figure 6). This approach is based on examining the slopes between all pairs of points, taking the median of the distribution as the final estimate. Kendall’s tau can then be used to determine if the estimated slope is significantly different from zero. This non-parametric method is robust against outliers and deals effectively with data heteroscedasticity (in this case, changes in the variance of firing rates across time). In five out of the six recording sessions, there was a significant trend for increasing firing rates from watch to imagine to attempt blocks (Kendall’s Tau *p* < 0.05). Importantly, this trend in EFR occurred even when the order of the blocks was reversed (note negative slope in Figure 6F). We also examined trends within each of the three blocks in each session. Seven out of the total of 18 blocks displayed a slope significantly different from zero. However, the changes within blocks did not correspond to the trends observed between blocks: five out of the seven blocks with significant slopes displayed changes in firing rate opposite to the between-block trend. Note that the magnitudes of the slopes were comparable across within and between block analysis. In summary, we observed changes in firing rate both within and across blocks. However, all significant effects across blocks indicated an increase in activity from W to I to A, while within block trends were not consistent. Our findings suggest that although there were random fluctuations in firing rates over time, these were combined with a consistent increase in cortical activity reflecting increased volitional intent.

## Discussion

This study demonstrates that the firing patterns of neurons in human motor cortex encode information related to sensory inputs and VoS in addition to movement features. Passively watching, mentally rehearsing, and attempting execution of movements activate shared populations of movement-related neurons. However, each of these VoSs results in a unique pattern of neural activity. Our findings go beyond indirect methods that monitor changes in general excitability by directly examining information present in single-unit spiking. Our results are in agreement with studies in NHPs, where robust activation of motor cortex during passive observation of actions has been previously reported (Wahnoun et al., 2006; Tkach et al., 2007; Dushanova and Donoghue, 2010; Vigneswaran et al., 2013). Primate studies have highlighted a class of neurons responding to both action execution and observation, termed “mirror neurons” (Fabbri-Destro and Rizzolatti, 2008). Recording from human participants allowed us to probe a wider range of VoSs by adding the “imagine” condition to “watch” and “attempt.” A shared subset of action-encoding neurons was engaged across VoS conditions. Approximately 14% of all neurons sampled displayed this property (more than a quarter of the action selective population). While about half of the action selective neurons were exclusively related to one state, the most reliable decoding results were obtained from neurons that were informative across multiple conditions (Figure 3). Action and VoS-related information was often combined at the level of single-units. Our results reveal a gradient of single-unit properties combining action and VoS information to various degrees, rather than discrete classes of neurons engaged by specific combinations of VoSs. This typed of “mixed selectivity” has also been reported for human parietal cortex, where single units have been shown to combine information reflecting VoS (attempt vs. imagine) and side of the body (right/left) within the context of a particular effector (hand squeeze or shoulder shrug; Zhang et al., 2017).

Jeannerod (2001) coined the term simulation states (“S-states”) to describe mental states that engage motor areas of the brain without resulting in overt movement. He proposed the existence of a “core” network that is activated during action execution and across all S-states, including mental rehearsal of actions (imagined actions) as well as action observation. The recruitment of additional neural circuitry in each case would result in distinct activity patterns unique to each condition within partially overlapping cortical networks. Mental imagery has been successfully used as a motor rehabilitation strategy after stroke, supporting the idea of overlapping cortical substrates (Page et al., 2001, 2007). Mukamel et al. (2010) have described overlapping activation patterns for single units during action observation and execution in human supplementary motor area, cingulate cortex and medial temporal lobe, lending further support to the concept of overlapping networks. Our results confirm that changing the VoS engages partially overlapping ensembles of neurons in human motor cortex, producing distinct activity patterns that vary across different reaching actions while simultaneously reflecting cognitive state. Our findings suggest that verbal instructions specifying a desired level of volitional engagement—the only external stimulus varied across VoS conditions in our task—can dramatically alter motor cortical activity. This finding provides a direct demonstration at the single neuron and ensemble level that cognitive and sensory signals engage motor cortex in the absence of performed actions, even when there is knowledge that the attempted action will not result in movement (a condition, notably, that can only be assessed when the participant is unable to move her limb). Our results reinforce the view that motor cortex is not just a final summing point for cerebral motor planning and execution, but part of a broader perception-to-action network engaged even when movement is not performed. These results indicate that brain computer interfaces (BCIs) relying on motor cortex will benefit from accounting for sensory and intentional signals that may otherwise be interpreted as noise. The unexpected depth of information present in motor cortex presents both a challenge and an opportunity for the development of BCIs.

The activity of single units was modulated across different reaching actions whether participants were instructed to watch, imagine, or attempt the movements (Figure 1). The most accurate decoding results were obtained during attempted movements, but action related information was consistently detected in all three conditions (Figure 4). Observed and imagined movements were associated with fewer active neurons and overall lower firing rates (Figures 2, 5, 6), in agreement with results obtained NHPs (Vigneswaran et al., 2013). This finding could also account for the deficits in decoding based on action observation reported by Waldert et al. (2015): local averaging in field potentials could result in decreased signal to noise ratios, even if the selectivity of individual neurons was comparable across conditions (Figure 3).

Although about half of the neurons displayed action selective responses in both participants, VoS-selective neurons were more than three times as abundant in participant S3 compared to T1. Only about one quarter of action selective neurons in T1 showed VoS effects, while nearly all action selective neurons showed VoS effects in S3. Participant S3 also presented more than twice the number of VoS-selective neurons with no action selectivity. The differences observed between the two participants could be related to underlying neuromotor pathology. Previous fMRI studies indicate that MI (but not prefrontal cortex) of people with ALS displays elevated activity during movement compared with controls with peripheral neuromuscular syndromes and healthy volunteers (Stanton et al., 2007; Li et al., 2015). However, these studies did not examine movement observation or imagined movement. Additionally, they did not include participants with brainstem stroke, making direct comparisons with our results difficult. Here, we observe a somewhat different pattern: our data shows that during attempted action T1 (ALS) had average activity levels comparable to S3 (brainstem stroke) but during watch and imagine, average firing rates were higher in T1 (Figure 6). Increased cortical excitability during imagined movements and passive observation could potentially mask the differences observed between VoSs. This could account for the increases similarity between W, I and A conditions in participant T1. Individual variations in the ability to perform motor imagery are an additional factor that could potentially influence our results (Marchesotti et al., 2016). The length of the recording electrodes (1.0 mm electrodes in T1, 1.5 mm electrodes in S3), and experience with BCI control (less than 2 months for T1, compared with more than 4 years for S3 at the beginning of the sessions) also differed between the two participants. Overall, participant S3 tended to have more accurate closed-loop BCI control (assessed during sessions not included in this study, see Jarosiewicz et al., 2013 for details). Differences in movement encoding have been shown based on the spike amplitude and quality of recorded units (Perel et al., 2015; Oby et al., 2016). However, differences in neural recording quality (reflected by SNR) are unlikely to underly the lower decoding accuracy for T1, since SNR values were actually higher in this participant (mean 2.35, SD 1.16) than for S3 (mean 1.72, SD 0.47). To determine if these factors contribute to the differences observed will clearly require substantially more study over a larger number of participants. However, despite very different underlying causes of tetraplegia, both participants showed effects of both VoS and movement kinematics on MI neural ensemble activity, suggesting that perceptual and cognitive contributions are a general feature of MI activation.

## Materials and Methods

### Participants

Permission for these studies was granted by the US Food and Drug Administration (Investigational Device Exemption. Caution: Investigational device. Limited by federal law to investigational use). This study was carried out with informed consent from all subjects. All subjects gave written informed consent in accordance with the Declaration of Helsinki. The protocol was approved by the Partners Healthcare/Massachusetts General Hospital Institutional Review Board. Participants who were unable to sign their name due to tetraplegia but who can speak may make a mark on the informed consent form that is witnessed by a family member and/or third-party witness. Participants who are unable to write and are unable to speak have sufficient eye movements to ask questions by choosing letters from an audibly recited alphabet or using an eye-gaze based assistive technology. In these instances, consent is further observed and attested to by a family member and third-party witness. The two participants in this study (S3 and T1) were enrolled in a pilot clinical trial of the BrainGate2 Neural Interface System^1^, and were implanted with a 96-channel intracortical silicon microelectrode array (Cyberkinetics Neurotechnology Systems, Inc., now Blackrock Microsystems, Salt Lake City, UT, USA), as previously described (Hochberg et al., 2006; Simeral et al., 2011).

At the time of this study, participant S3 was a 58-year-old woman with tetraplegia and anarthria (inability to speak) resulting from a pontine stroke that occurred 9 years prior to array implantation. She retained eye movement, some head movement, and facial expression and breathes spontaneously. She had bilateral upper extremity flexor spasms that occur sporadically with many intended body movements. The array, which had electrodes 1.5 mm in length, was implanted in the hand area of her dominant motor cortex (see Simeral et al., 2011; Hochberg et al., 2012 for additional detail). Participant T1 was a 48-year-old woman with tetraplegia resulting from ALS, diagnosed 6 years prior to array implantation. She was completely paralyzed except for some eye movement, and her breathing was assisted by a ventilator. The array, with electrodes 1.0 mm in length, was implanted in her dominant motor cortex.

### Neural Recording

Neural activity was detected by the 96-channel microelectrode array and monitored via a cable that was connected to a percutaneous connector during each 2–3 h recording session. Signals recorded at 30 KHz were filtered offline (4th order non-causal Butterworth, low cutoff  250 Hz, high cutoff  7,500 Hz), coincident noise in the raw signal was reduced using common-average referencing: the 80 channels with the lowest mean root-mean-squared (RMS) voltage value were averaged and subtracted from all channels. For each channel, 1.6 ms spike waveforms were extracted using a 4RMS threshold with a 1 ms lockout. Differences in spike waveform shape were used to identify single-unit activity using custom-made software employing template matching with spike overlap resolution (Vargas-Irwin and Donoghue, 2007). Only units exceeding a signal to noise ratio (SNR) of 1.2 were included in the analysis (SNR = mean spike amplitude/95% confidence interval for the distribution of voltages during non-spiking periods). Note that the sets of neurons recorded from day to day using microelectrode arrays can potentially overlap. Fraser and Schwartz (2012) reported that ~50% of neurons in monkey motor cortex were stable over a period of 2 weeks. Recording sessions for each participant should therefore be considered as partially overlapping samples of the total pool of cortical neurons.

### Task Details

For each session, the following instructions were read prior to the relevant block by a clinical technician:

- Watch: “You are about to be shown a series of movies of an arm performing several different motions. Please try to watch the arm without making any association to it. You should remain relaxed and motionless and watch the movies as if they are just a series of animations on the screen. Do not imagine yourself moving and do not attempt to move as depicted, but focus on the screen and watch the animation.”
- Imagine: “You are about to be shown a series of movies of an arm performing several different motions. Imagine performing the same movement as the movie is played. Here we are testing your ability to imagine moving without intending to move. You should remain relaxed and think about how it looks to make the movement and how it feels. It is important that you only imagine these movements, and not attempt to actually make them. As a reminder, the example given earlier was that you could imagine moving your eyes to the left or actually move them to the left. It might help to think of imagining in this case as “mentally practicing” a movement. If you need to be reminded of the difference between imagining moving and moving, please indicate so now.”
- Attempt: “You are about to be shown a series of movies of an arm performing several different motions. Please try to watch the arm and attempt to make the same movements along with it. It is important that you actually attempt to perform the same movements. Do your best to keep pace with the movies regardless of your actual movements.”

Animated movies representing center-out movements to four peripheral targets (45°, 135°, 225° and 315° in the vertical plane) from a first-person perspective were generated using Poser software (SmithMicro). The movies were viewed on an LCD monitor (46.26 cm). Participants are seated 57 cm away from the center of the monitor, subtending a visual angle of ~1°/cm. Each instruction block consisted of a random sequence of the four movies (reaching in four directions) with 10 repetitions of each (40 movies total). The same movies were used for all instruction conditions. A block design was adopted to make it easier for participants to shift between W, I and A conditions. One 40-trial block of each type was presented during each of the six sessions (three per participant). For participant S3, the order of the blocks was always W, I, A. The same order was used for the first two sessions with participant T1, while the last session used the reverse order (A, I, W).

S3 was observed intermittently to generate spastic flexion movements about the elbow. These movements would occur, at some moments but not others, when she was Attempting movements, but not when Imagining or Watching movements. While possible that these intermittent movements yielded proprioceptive feedback that changed firing rates in recorded neurons, the intermittent nature of this event would only have affected the magnitude, but not the presence, of some of the differences in VoS and action reported with Attempt epochs.

### SSIMS

Spike train similarity is defined in terms of how much individual spikes have to be shifted in time, added, or removed to make two spike trains match precisely (Victor and Purpura, 1996, 1997; Victor, 2005). Each operation is assigned a cost: 1 for addition or deletion and Δt*q for shifting. The value of 1/q sets the balance between shifting a spike in time vs. addition and deletion operation. For the data analysis presented, the 1/q temporal actuary setting was equivalent to 100 ms (so that shifting a spike by more than 100 ms was equivalent to inserting/deleting a spike). The distance between two spike trains is defined as the (minimum) sum of the set of operations separating them. The activity pattern recorded during a single trial is represented in terms of similarity to all other trials in the dataset (that is to say, as a vector of pair-wise distances). This representation is then transformed using a form of non-linear dimensionality reduction (van Der Maaten and Hinton, 2008), resulting in a low dimensional representation of the differences between trials. Thus, the distance between two points in this projection space represents the similarity between the two activity patterns. Two identical firing patterns correspond to the same point in this space; the more different they are, the farther apart they are when in the SSIMS projection.

We used SSIMS for two kinds of statistical tests. For single units, we determined if trials within a given experimental conditon (i.e., VoS or action) were more similar to each other than to trials in other conditions by comparing the distributions of pair-wise distances in the SSIMS projection (Kruskal-Wallis *p* < 0.05). This approach detects whether certain types of trials are more likely to cluster together (i.e., be more similar), reflecting category-related information. SSIMS allowed us to perform these comparisons over a relatively large time window (4.5 s window starting 200 ms after movement onset) while taking into account the timing of each spike, resulting in greater sensitivity that rate based methods. For example, since each video included movement towards a peripheral target and back to the center (i.e., movement in opposite directions), action-related information tended to be washed out if only the firing rate was taken into account. Adjusting the analysis time window could address this problem, but decreasing the ammount of data examined ultimately resulted in fewer action-related neurons being detected.

We also used SSIMS to generate discrete classifiers for action and VoS conditions, using either single units (Figure 3) or ensembles (Figure 4). Classification accuracy was used as a way to quantify the information present in the neural activity patterns. Classification was performed by projecting neural activity patterns (over the full set of neurons) onto a 15-dimensional SSIMS representation generated using data from a 4.5 s window starting 200 ms after movement onset. We used a nearest-neighbor classifier to decode movement targets. Decoding accuracy was evaluated using leave-one-out cross validation: each trial was assigned to the target type of the most similar (closest) neighbor in the SSIMS projection. The expected value and 95% confidence interval of the chance distribution for classification was calculated using 10,000 iterations with randomly shuffled trial labels.

### State Change Index (SChI)

To determine whether a change in firing rate occurred at time t, we first counted the spikes in two 400 ms windows before and after the chosen time for each trial. We then selected a random subset of the trials (80%) and tested whether spike counts before and after t were drawn from a statistically different distribution (KS *p* < 0.01). This evaluation of subsampled sets was repeated 1,000 times in order to test across trial reliability. The state change index (SChI) at time t is defined as the fraction of subsample sets in which there was a statistically significant difference between the two bins. Under the null hypothesis that firing rates are drawn from the same distribution, we would expect the SChI to be less than the set alpha value of 0.01 (representing 10 out of 1,000 shuffles). SChI values >0.02 (twice the expected chance value) were considered to represent a significant change in firing rate. We reported the latency in the response of each unit as the time of the first window with significant SChI.

## Author Contributions

JF, BK, LH and JD conceived of the research question and designed the experiments. BK coded the movies displayed to the participants. JS, BS, EO, JF and BK refined the experimental protocol and collected the data. CV-I, BK and JF analyzed the data, with input from LH and JD. CV-I drafted the manuscript, which was further edited by all authors. SC is a clinical co-investigator of the pilot clinical trial and assisted in the clinical oversight of the participants. EE and GF planned and executed the electrode array implants. JD and LH conceived of and planned the ongoing BrainGate research. LH is the Investigational Device Exemption Sponsor-Investigator and directs the BrainGate2 pilot clinical trial.

## Conflict of Interest Statement

The authors declare that the research was conducted in the absence of any commercial or financial relationships that could be construed as a potential conflict of interest.

## Footnotes

^1^www.clinicaltrials.gov/ct2/show/NCT00912041
